# Gluten measurement and its relationship to food toxicity for celiac disease patients

**DOI:** 10.1186/1746-4811-4-26

**Published:** 2008-10-28

**Authors:** Diane R Lester

**Affiliations:** 1 School of Plant Science, University of Tasmania, Private bag 45, Hobart 7001, Australia

## Abstract

The gluten analysis of foods has long had limitations, which have precluded food standards authorities from issuing standards for gluten-free foods based on final gluten content. The Codex Alimentarius and the Food and Drug Administration have taken steps towards such standards in which they favour the R5-enzyme-linked immunosorbent assay for gluten analysis. If this method is to be widely employed, its limitations should be recognised. Above all, it should be noted the ability of R5-enzyme-linked immunosorbent assay, and other methods, to measure gluten's toxicity toward celiac disease patients is not validated clinically. Gluten is a complex mixture of proteins and its toxicity is not fully understood. Analytical methods are a valuable tool in the definition of gluten-free foods, but they should be employed with appropriate caveats in ensuring the safety of the foods.

## Introduction

Celiac disease (CD) is chronic gastrointestinal inflammation caused by an aberrant immune response to dietary gluten [1]. It is treated by life-long adherence to a gluten-free (GF) diet.

Speciality GF foods cater for CD patients. ELISA assays have long been used to monitor these foods, but the methods have had limitations which, until recently, have prevented them gaining acceptance as the basis for official GF standards [2,3]. The analysis of gluten is challenging because gluten is a mixture of water-insoluble proteins, derived from wheat, barley or rye grain, which in commercial foods, is within a range of matrices and is modified variously by heat and processing [4].

Commercial methods of gluten analysis are based on the ELISA, employing an initial extraction step to solubilise gluten from food samples. The limitations and efficiencies of these methods for the testing of GF foods were reviewed previously [4].

New improved gluten ELISAs have since appeared on the market [5,6] and others are in development [7,8]. The commercial R-ELISA [5] has been deemed sufficiently reliable and sensitive to support standards for GF foods based on final gluten content [9]. If the method is to be widely employed, its limitations should be recognised so the method is used correctly.

Herein, I critically examine the basis of the R5-ELISA and other recent ELISA methods of gluten analysis.

## Discussion

### Defining gluten

Gluten is a scientifically imprecise term and its definition varies, even when GF foods are concerned. Herein, the current Codex definition of gluten is employed, which is 'a protein fraction from wheat, rye, barley, oats or their crossbred varieties and derivatives thereof, to which some people are intolerant and that is insoluble in water and 0.5 M NaCl' [9]. This definition is similar to that recently proposed by the FDA, 'the proteins that naturally occur in a prohibited grain and that may cause adverse health effects in persons in celiac disease' [10].

Alternatively, gluten is sometimes defined by its solubility only, a feature which derives from the high proline and glutamine content of the native gluten proteins. For example, 'a protein fraction of wheat, rye or barley insoluble in water or a solution of 0.5 M NaCl' [4]. Such a definition is not used here because it does not support meaningful discussions concerning food safety. (Some food processing procedures increase gluten's solubility, but do not necessarily diminish the protein fraction's harmful effect).

### Demonstrating gluten's toxicity

Demonstrating intolerance to gluten is a complex endeavour. The toxicity of gluten in CD stems from an immune response involving both innate and adaptive systems [1]. No model is available to replicate the response, although rhesus macaques were recently proposed [11]. The demonstration of gluten intolerance depends on *in vivo *challenge studies.

CD has a highly variable presentation [1] and symptoms are considered unreliable as an indicator of active disease. The defining indicator of gluten-induced damage in CD is histopathology of the mucosa of the small intestine [12]. It only develops in response to ongoing gluten exposure, which means the investigation of gluten intolerance faces design and ethical hurdles. Other factors associated with CD hinder representative studies. They include the heterogeneous presentation of disease, a high rate of under-diagnosis [1] and the lifestyle challenges of a truly GF diet [13].

### Defining gluten-free foods

In many circles, a zero tolerance approach to gluten in GF foods is considered impractical. With derivatives of wheat, and to a lesser extent barley, used widely in mainstream food channels, GF foods are susceptible to contamination, even when produced in dedicated facilities. Some GF foods in Europe are even based on wheat-starch [3] which, though processed to remove gluten, contains gluten residues. However, many GF foods are entirely free of gluten-containing grains [14] and they are available throughout Europe. Preventative measures to minimise contamination are employed by GF manufacturers which, in some cases, are very strict. For example, a core group of manufacturers not only exclude all gluten-containing grains from their foods and use dedicated facilities, they also heed the provenance of all their ingredients.

In efforts to reach a universal definition of GF foods, studies have aimed to define safe threshold gluten for CD patients through *in vivo *challenge studies. While it remains limited, the evidence base in this area has strengthened in recent years [15]. It appears the extent to which gluten must be excluded from the diet varies between CD individuals [12,15] with highly sensitive individuals difficult to study. They do not tolerate [12], and are possibly deterred by, an extended gluten challenge.

Guidelines for specialty GF foods vary between regulatory authorities. The FDA recently proposed draft GF standards for the first time, which are based on final gluten content [10]. Codex is in the final stages of approving similar standards [2,9]. The draft standards permit trace gluten in GF foods, but aim to keep total dietary gluten well below levels generally accepted as safe for CD patients. They allow ingredients derived from gluten-containing grains in the foods, providing the ingredients have been processed to remove gluten and prescribed limits are met.

In addition, Codex has had standards since 1981, which define GF foods according to the nitrogen content of raw ingredients [16]. These standards are only applicable to GF foods with ingredients derived from wheat, barley or rye.

The Codex has endorsed the R5-ELISA as a means of upholding GF standards based on final gluten content [9]. The FDA has tentatively endorsed the method and acknowledges that future methods including other ELISAs, may prove useful in the area [10]. In light of such significant endorsement, it is important to consider the remaining limitations of gluten analysis by ELISA.

### The basis for gluten's toxicity

*In vitro*, and to a lesser extent, *in vivo *methods have been useful in dissecting the basis for gluten's immunotoxicity. Activation of CD4(+) T cells in the small intestinal mucosa by gluten peptides released by digestive enzymes is a key event in CD [17]. A direct effect from other gluten peptides on the intestinal epithelium has a role in inflammation [17]. Autoantibodies are associated with active CD and their role in pathogenesis is currently an active area of research, eg. [18].

Multiple peptides are implicated in T cell stimulation, present in both major fractions of gluten (in wheat these fractions are the gliadins and glutenins) [17]. A single peptide located within a region resistant to digestive enzymes is the immunodominant portion of the gluten subfraction, α-gliadin, at least in adult patients [19,20]. The full potency of this peptide, and that of others, is dependent on its modification by a tissue transglutaminase within the intestinal mucosa. The enzyme introduces a negative charge into the peptide, which enhances class II MHC binding on antigen-presenting cells.

The crucial role of T cell activation in disease pathogenesis is evident from the association of CD with class II MHC genotype. Over 90% of patients are positive for the HLA DQ2 heterodimer with the remainder positive for HLA DQ8 [17]. The peptides stimulatory to T cells in HLA DQ8 individuals appear to be distinct from those of the HLA DQ2 model [21].

### The bases of recent gluten ELISAs

The commercial R5-ELISA is hailed as a significant advance in the area of gluten detection [22]. Nonetheless, developments in the area continue. The bases of the R5-ELISA and other recent methods are examined here. Earlier gluten ELISAs are not included because they are already appraised in detail [4].

The R5-ELISA [5] and subsequent methods [7,8] are based on monoclonal antibodies which target sequences characteristic of peptides stimulatory to T cells. The latest methods [7,8] have highest specificity for gliadin peptides of known immunodominance in HLA DQ2 patients, in particular the well-characterised α-gliadin peptide. One method also has demonstrated cross-reactivity with a gliadin peptide which invokes an innate immune response [8].

The strategy of targeting immunogenic peptides with a gluten ELISA aims for an analytical method, which acts as an indicator of food toxicity to CD patients. It contrasts with the strategy of an earlier, but widely-used gluten ELISA [23] which targets the most heat-stable subfraction of gliadin, the ω-gliadins.

It should be noted the gluten proteins are not necessarily intact when present in food. The original version of the R5-ELISA and the anti-ω-gliadin ELISA employ a sandwich format, which is unable detect small gluten fragments [24]. The latest R5-ELISA, together with other recent methods, employs a competitive format, which enables detection of gluten peptides containing single epitopes and therefore is suitable against highly degraded protein. At least in sandwich form, the R5-ELISA is considered more sensitive than the anti-ω-gliadin ELISA and better at detecting barley gluten [2,24]. An inability to detect barley and rye sequences is a weakness of another recently-developed commercial gluten ELISA [6,25].

Though commercially available, the competitive R5-ELISA is not yet trialled as extensively as the sandwich R5-ELISA. The abilities of the sandwich R5-ELISA and the anti-ω-gliadin to detect the gluten proteins in food were reviewed recently, and the merits of the competitive R5-ELISA considered [24].

All gluten ELISAs use a standard of native gliadin [3,4]. The contribution of the glutenins toward gluten measurements is estimated, based on the assumption that both fractions of gluten are present in equal amounts.

### The relationship between gluten measurements and the toxicity of food to CD patients

It should be noted that the relationship between ELISA measurements of gluten in food and the toxicity of the foods toward CD patients is not directly investigated. No gluten ELISA has been evaluated through clinical trials. The relationship has been explored to a limited extent with a bioassay employing T cells isolated from CD patients [7].

Thus, the capabilities of ELISA methods to ensure the GF status of foods are assumed, combining analytical estimations of gluten in food with knowledge on daily safe threshold levels of gluten and current understanding of its toxicity toward CD patients.

### Remaining limitations of gluten ELISAs

Notwithstanding the advantages of recent methods, the assessment of the safety of GF foods for CD patients based on gluten analysis by ELISA is not clearcut. The multiple challenges [4] associated with the testing of GF foods are not completely surmounted by the methods. Uncertainties remain regarding (1) the quantitative measurement of gluten in foods and (2) analytical-based predictions of food toxicity to CD patients.

Perhaps the most intractable aspect of gluten analysis is the accurate standardisation of measurements. The most soluble half of wheat gluten, gliadin, has been deemed the best standard for gluten measurements, however it may not accurately represent gluten in food in every instance.

Gluten's precise composition varies between species and cereal variety. In addition, the food industry uses technological procedures in the preparation of ingredients and foods, which modify gluten physically, chemically or enzymatically. These modifications, as well as matrix effects associated with food ingredients, may affect gluten's solubility, its intermolecular associations, not to mention the availability, and even the sequence, of its epitopes. These factors conceivably impact on gluten analysis by ELISA protocols, during extraction and/or immunodetection. To illustrate the vagaries associated with the standardisation of gluten measurements, gliadin is no longer considered ideal as a standard for measuring barley-derived gluten with the R5-ELISA [24].

It is practically impossible to analyse the gluten of commercial foods in a controlled fashion because the foods are so various and their exact composition is unknown. In trials of the R5-ELISA [5,26], thousands of commercial GF foods were analysed, their gluten content estimated against a pure native gliadin standard. The validity of this approach in establishing the method's reliability can be challenged because the approach does show whether gluten in the foods evades detection in some instances. Nor is it able to reveal whether non-specific antibody interactions are occurring and leading to a skewing of ELISA values. Controlled experiments were included in the trials [5], however they involved relatively simple foods spiked with native gliadin only, which, arguably, do not reflect the diversity of commercial foods.

Indeed, a subsequent study [25] suggests that the R5-ELISA fails to detect some forms of gluten, at least when employed in the sandwich format. The sandwich R5-ELISA yielded lower gluten values on beer samples than the competitive R5-ELISA. This difference was attributed to an inability of the sandwich R5-ELISA to detect hydrolysed gluten, however the experiments were insufficiently controlled to show that the competitive R5-ELISA reliably detected all gluten in the commercial samples tested.

The use of a gliadin standard for gluten measurements may also be challenged on the basis it overlooks the glutenin fraction of gluten. Formerly, the contribution of this less soluble fraction to gluten toxicity was unclear [4], however glutenin peptides are now known to be involved in T cell activation in CD patients [17]. Being highly insoluble, the glutenins may persist throughout processing procedures, which eliminate the gliadins [27]. Thus, it is debatable whether a gluten detection method is capable of indicating food toxicity, using a gliadin standard only.

Other weaknesses can be found in the theory behind gluten ELISAs. Gluten's toxicity is related to its quality as well as its quantity. The immunogenicity of gluten proteins varies according to influences such as cereal variety and food processing procedures [2]. Most notably, deamidation has implications for food safety [28]. Deamidated gluten is detected inefficiently by the R5-ELISA [29], yet may have heightened toxicity in some instances.

## Conclusion

Gluten ELISAs are able to detect gluten contamination in foods in many instances and are a valuable tool in the analysis of GF foods. However, they are not clinically validated as an indicator of food toxicity toward CD patients and analytical-based predictions of food toxicity are not straightforward. The methods cannot on their own ensure the safety of GF foods, but must be employed with appropriate caveats. False reliance on the methods is to be avoided, if official standards for GF foods based on final gluten content are widely adopted.

## Abbreviations

CD: celiac disease; Codex: World Health Organization/Food and Agriculture Organization of the United Nations, Codex Alimentarius Commission; ELISA: enzyme-linked immunosorbent assay; FDA: Food and Drug Administration; GF: gluten-free; HLA: human leucocyte antigen; MHC: major histocompatibility complex

## Competing interests

The author declares that she has no competing interests.

## References

[B1] Green PH, Cellier C (2007). Celiac disease. N Engl J Med.

[B2] Troncone R, Auricchio R, Granata V (2008). Issues related to gluten-free diet in coeliac disease. Curr Opin Clin Nutr Metab Care.

[B3] Hischenhuber C, Crevel R, Jarry B, Maki M, Moneret-Vautrin DA, Romano A, Troncone R, Ward R (2006). Review article: safe amounts of gluten for patients with wheat allergy or coeliac disease. Aliment Pharmacol Ther.

[B4] Denery-Papini S, Nicolas Y, Popineau Y (1999). Efficiency and limitations of immunochemical assays for the testing of gluten-free foods. J Cereal Sci.

[B5] Mendez E, Vela C, Immer U, Janssen FW (2005). Report of a collaborative trial to investigate the performance of the R5 enzyme linked immunoassay to determine gliadin in gluten-free food. Eur J Gastroenterol Hepatol.

[B6] Gabrovska D, Rysova J, Filova V, Plicka J, Cuhra P, Kubik M, Barsova S (2006). Gluten determination by gliadin enzyme-linked immunosorbent assay kit: interlaboratory study. J AOAC Int.

[B7] Spaenij-Dekking EH, Kooy-Winkelaar EM, Nieuwenhuizen WF, Drijfhout JW, Koning F (2004). A novel and sensitive method for the detection of T cell stimulatory epitopes of alpha/beta- and gamma-gliadin. Gut.

[B8] Moron B, Bethune MT, Comino I, Manyani H, Ferragud M, Lopez MC, Cebolla A, Khosla C, Sousa C (2008). Toward the assessment of food toxicity for celiac patients: characterization of monoclonal antibodies to a main immunogenic gluten peptide. PLoS ONE.

[B9] Codex Alimentarius Commission (2008). Draft revised standard for gluten-free foods, Step 8. ALINORM 08/31/26.

[B10] US Department of Health and Human Services; Food and Drug Administration (2007). Food labeling; Gluten-free labelling of foods. Proposed rule. Fed Reg.

[B11] Bethune MT, Borda JT, Ribka E, Liu MX, Phillippi-Falkenstein K, Jandacek RJ, Doxiadis GG, Gray GM, Khosla C, Sestak K (2008). A non-human primate model for gluten sensitivity. PLoS ONE.

[B12] Catassi C, Fabiani E, Iacono G, D'Agate C, Francavilla R, Biagi F, Volta U, Accomando S, Picarelli A, De Vitis I, Pianelli G, Gesuita R, Carle F, Mandolesi A, Bearzi I, Fasano A (2007). A prospective, double-blind, placebo-controlled trial to establish a safe gluten threshold for patients with celiac disease. Am J Clin Nutr.

[B13] Leffler DA, Edwards George JB, Dennis M, Cook EF, Schuppan D, Kelly CP (2007). A prospective comparative study of five measures of gluten-free diet adherence in adults with coeliac disease. Aliment Pharmacol Ther.

[B14] Zhang J (2007). FDA calls for standards on gluten; A growing number of consumers seeks to avoid the protein. Wall Street Journal New York.

[B15] Akobeng AK, Thomas AG (2008). Systematic review: tolerable amount of gluten for people with coeliac disease. Aliment Pharmacol Ther.

[B16] Codex Alimentarius Commission (1981). Standard for "Gluten-free foods". CODEX STAN 118-1981 (Amended 1983).

[B17] van Heel DA, West J (2006). Recent advances in coeliac disease. Gut.

[B18] Caputo I, Barone MV, Martucciello S, Lepretti M, Esposito C (2008). Tissue transglutaminase in celiac disease: role of autoantibodies. Amino Acids.

[B19] Arentz-Hansen H, Korner R, Molberg O, Quarsten H, Vader W, Kooy YM, Lundin KE, Koning F, Roepstorff P, Sollid LM, McAdam SN (2000). The intestinal T cell response to alpha-gliadin in adult celiac disease is focused on a single deamidated glutamine targeted by tissue transglutaminase. J Exp Med.

[B20] Anderson RP, Degano P, Godkin AJ, Jewell DP, Hill AV (2000). In vivo antigen challenge in celiac disease identifies a single transglutaminase-modified peptide as the dominant A-gliadin T cell epitope. Nat Med.

[B21] Henderson KN, Tye-Din JA, Reid HH, Chen Z, Borg NA, Beissbarth T, Tatham A, Mannering SI, Purcell AW, Dudek NL, van Heel DA, McCluskey J, Rossjohn J, Anderson RP (2007). A structural and immunological basis for the role of human leukocyte antigen DQ8 in celiac disease. Immunity.

[B22] Stern M (2005). A major step towards a practical and meaningful gluten analysis. Eur J Gastroenterol Hepatol.

[B23] Skerritt JH, Hill AS (1991). Enzyme immunoassay for determination of gluten in foods: collaborative study. J Assoc Off Anal Chem.

[B24] Thompson T, Mendez E (2008). Commercial assays to assess gluten content of gluten-free foods: why they are not created equal. J Am Diet Assoc.

[B25] Dostalek P, Hochel I, Mendez E, Hernando A, Gabrovska D (2006). Immunochemical determination of gluten in malts and beers. Food Addit Contam.

[B26] Valdes I, Garcia E, Llorente M, Mendez E (2003). Innovative approach to low-level gluten determination in foods using a novel sandwich enzyme-linked immunosorbent assay protocol. Eur J Gastroenterol Hepatol.

[B27] Wang JS, Zhao MM, Zhao QZ, Bao Y, Jiang YM (2007). Characterization of hydrolysates derived from enzymatic hydrolysis of wheat gluten. J Food Sci.

[B28] Malandain H (2005). Transglutaminases: a meeting point for wheat allergy, celiac disease, and food safety. Eur Ann Allergy Clin Immunol.

[B29] Kahlenberg F, Sanchez D, Lachmann I, Tuckova L, Tlaskova H, Mendez E, Mothes T (2006). Monoclonal antibody R5 for detection of putatively coeliac-toxic gliadin peptides. Eur Food Research Technol.

